# Urban colonization through multiple genetic lenses: The city‐fox phenomenon revisited

**DOI:** 10.1002/ece3.4898

**Published:** 2019-01-31

**Authors:** Alexandra L. DeCandia, Kristin E. Brzeski, Elizabeth Heppenheimer, Catherine V. Caro, Glauco Camenisch, Peter Wandeler, Carlos Driscoll, Bridgett M. vonHoldt

**Affiliations:** ^1^ Department of Ecology and Evolutionary Biology Princeton University Princeton New Jersey; ^2^ School of Forest Resources and Environmental Science Michigan Technological University Houghton Michigan; ^3^ Department of Evolutionary Biology and Environmental Studies University of Zurich Zurich Switzerland; ^4^ Natural History Museum Fribourg Switzerland; ^5^ Laboratory of Comparative Behavioral Genomics National Institute on Alcohol Abuse and Alcoholism, National Institutes of Health Rockville Maryland

**Keywords:** colonization, contemporary evolution, genetic drift, major histocompatibility complex, selection, urbanization

## Abstract

Urbanization is driving environmental change on a global scale, creating novel environments for wildlife to colonize. Through a combination of stochastic and selective processes, urbanization is also driving evolutionary change. For instance, difficulty in traversing human‐modified landscapes may isolate newly established populations from rural sources, while novel selective pressures, such as altered disease risk, toxicant exposure, and light pollution, may further diverge populations through local adaptation. Assessing the evolutionary consequences of urban colonization and the processes underlying them is a principle aim of urban evolutionary ecology. In the present study, we revisited the genetic effects of urbanization on red foxes (*Vulpes vulpes*) that colonized Zurich, Switzerland. Through use of genome‐wide single nucleotide polymorphisms and microsatellite markers linked to the major histocompatibility complex (MHC), we expanded upon a previous neutral microsatellite study to assess population structure, characterize patterns of genetic diversity, and detect outliers associated with urbanization. Our results indicated the presence of one large evolutionary cluster, with substructure evident between geographic sampling areas. In urban foxes, we observed patterns of neutral and functional diversity consistent with founder events and reported increased differentiation between populations separated by natural and anthropogenic barriers. We additionally reported evidence of selection acting on MHC‐linked markers and identified outlier loci with putative gene functions related to energy metabolism, behavior, and immunity. We concluded that demographic processes primarily drove patterns of diversity, with outlier tests providing preliminary evidence of possible urban adaptation. This study contributes to our overall understanding of urban colonization ecology and emphasizes the value of combining datasets when examining evolutionary change in an increasingly urban world.

## 1 INTRODUCTION

Urbanization is increasing worldwide and broadly affecting wildlife across the growing rural–urban gradient (Knapp, Winter, & Klotz, 2017; Magle, Hunt, Vernon, & Crooks, 2012). Impacts of urbanization include homogenizing habitat and reducing species diversity (McKinney, 2006), reducing phylogenetic richness (Knapp et al., 2017), and increasing human–wildlife conflict (Soulsbury & White, 2015). Although urbanization can homogenize a multispecies wildlife community, urban gradients can lead to population subdivision within a species due to reduced gene flow across urban barriers and divergent selection pressures between rural and urban conspecifics (Johnson & Munshi‐South, 2017; Santangelo, Ruth Rivkin, & Johnson, 2018). Urban barriers include buildings, freeways, and densely populated areas (such as city centers), with divergent selection pressures including varied noise and light pollution, diet, and disease and toxicant exposure between urban and rural environments (Brans, Stoks, & De Meester, 2018; Harris & Munshi‐South, 2017; Isaksson, 2015; Ouyang et al., 2017; Sih, Ferrari, & Harris, 2011). The possibility for urban–rural gradients facilitating divergence has created a need for more research dedicated to assessing the evolutionary changes associated with urbanization (Alberti, 2015; Donihue & Lambert, 2015; Santangelo et al., 2018). As such, there has recently been a surge of studies examining the genetic effects of urbanization across diverse taxa, with each system providing unique insights into the processes shaping wildlife in the Anthropocene (Combs, Byers et al., 2018; Johnson, Prashad, Lavoignat, & Saini, 2018; Miles, Dyer, & Verrelli, 2018; Mueller, Kuhl et al., 2018; Theodorou et al., 2018).

Genetic drift and local adaptation are two forces that can influence population divergence in rural–urban conspecifics. For instance, habitat fragmentation in human‐modified landscapes can decrease gene flow across the rural–urban gradient, drive genotypic differentiation, and increase the impact of genetic drift in small, isolated urban populations (Keyghobadi, 2007; Rivera‐Ortíz, Aguilar, Arizmendi, Quesada, & Oyama, 2015; Zipperer, Foresman, Walker, & Daniel, 2012). Differentiation has been observed across taxa, including less vagile eastern red‐backed salamanders (*Plethodon cinereus*) in Montreal (Noël, Ouellet, Galois, & Lapointe, 2007), white‐footed mice (*Peromyscus leucopus*) in New York City (Munshi‐South & Kharchenko, 2010; Munshi‐South, Zolnik, & Harris, 2016), and highly mobile bobcats (*Lynx rufus*) and coyotes (*Canis latrans*) separated by a freeway in southern California (Delaney, Riley, & Fisher, 2010; Riley et al., 2006).

Urban colonization further constitutes a founder event, whereby decreased genetic variation may follow reduced effective population size (Greenbaum, Templeton, Zarmi, & Bar‐David, 2014; Nei, Maruyama, & Chakraborty, 1975). This phenomenon is observed during colonization of natural (Fabbri et al., 2007) and urban (DeCandia et al., 2019; Evans et al., 2009) areas, but may be exacerbated by the difficulty of navigating the urban matrix. If effective population size remains small through colonization and establishment, the resultant diversity loss may threaten long‐term viability due to increased risk of inbreeding (Frankham, 2005, 2008; Oakley, 2013; Spielman, Brook, Briscoe, & Frankham, 2004). Deleterious alleles may accumulate and decrease the fitness of urban colonizers. This diversity loss is especially problematic for threatened or sensitive species colonizing environments characterized by novel stressors, such as cities, where local adaptation may be necessary for persistence.

Despite decreases in effective population size, selection can maintain diversity during urban colonization and increase the frequency of adaptive alleles in urban populations (Donihue & Lambert, 2015). For example, immunogenetic variation in the major histocompatibility complex (MHC) gene family remained stable in urban colonizing dark‐eyed juncos (*Junco hyemalis*) amid losses of neutral variation (Whittaker, Dapper, Peterson, Atwell, & Ketterson, 2012). A similar pattern emerged in urban bobcats following a severe mange outbreak exacerbated by anticoagulant rodenticide exposure (Serieys, Lea, Pollinger, Riley, & Wayne, 2015). In these and other systems, balancing selection maintained diversity at MHC genes (Ferrer‐Admetlla et al., 2008); perhaps due to the selective advantage immunogenetic variation confers against disease risk (DeCandia, Dobson, & vonHoldt, 2018; Spielman et al., 2004).

In some cases, urban areas may even serve as “theaters for evolution,” where novel selective pressures drive rapid adaptation (Littleford‐Colquhoun, Clemente, Whiting, Ortiz‐Barrientos, & Frère, 2017). For example, signatures of urban adaptation have been reported for genes associated with lipid and carbohydrate metabolism (Harris & Munshi‐South, 2017), harm avoidance behavior (Mueller, Partecke, Hatchwell, Gaston, & Evans, 2013), and toxicant exposure (Reid et al., 2016). Thus, adaptation is a potent force that can influence divergence in rural–urban conspecifics during initial colonization and longer‐term persistence. Teasing its effects apart from drift through examination of genome‐wide SNPs and functionally linked markers will help determine how selection versus stochastic events shape wildlife populations across diverse rural–urban gradients.

**Figure 1:**
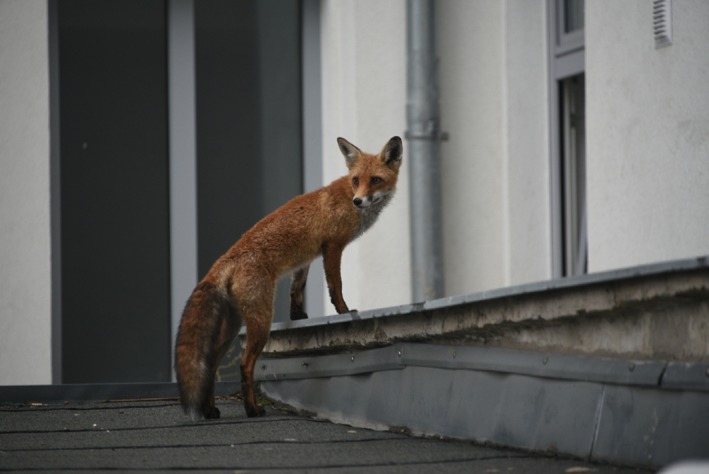
Red foxes (*Vulpes vulpes*) have successfully colonized urban areas in Europe since the 1930s. Photo credit: © L. Hamelbeck‐Galle/stadtwildtiere.at

One of the best documented urban wildlife colonizers has been the red fox (*Vulpes vulpes*; Figure 1), a generalist species that thrives across rural–urban gradients around the globe (Coman, Robinson, & Beaumont, 1991; Harris, 1981; Marks & Bloomfield, 1999). Red fox urbanization is most pronounced in Europe, where in Switzerland, city foxes dramatically increased in density during the mid‐1980s following successful eradication of rabies (Gloor, Bontadina, Hegglin, Deplazes, & Breitenmoser, 2001). Though prophylactic culling continued until 1995, release from rabies enabled fox populations to expand in rural and urban environments, with a large breeding population of more than 10 adult foxes per km^2^ detected in Switzerland's largest city, Zurich (Gloor et al., 2001). Mortality records and resident surveys reported a fourfold increase in fox sightings between 1985 and 2000, as family groups established territories throughout the Zurich metropolitan area (Gloor, 2002; Gloor et al., 2001). Further analysis of movement patterns revealed that foxes settled in rural or urban areas remained almost exclusively within one habitat type, despite proximity to the rural–urban border (Gloor, 2002). This restricted movement suggested the existence of subpopulations within Zurich's urban and adjacent rural areas.

To explore the genetic consequences of these observations, Wandeler, Funk, Largiadèr, Gloor, and Breitenmoser (2003) used 11 neutral microsatellite loci to report on patterns of genetic diversity five to seven generations after initial colonization (with an assumed generation time of two to three years). As predicted in founder events, urban foxes exhibited decreased variation and significant genotypic differentiation when compared to rural foxes, a result supported by previous radio‐tracking data (Gloor, 2002). Further, pairwise *F*_ST_ was highest (*F*_ST_ = 0.068 ± 0.020) and immigration rates lowest between the two urban subpopulations, suggesting that Lake Zurich and the Limmat River in the Zurich city center presented substantial isolating barriers for urban foxes. The authors concluded that fox colonization of Zurich likely occurred through two founder events (one east and one west of Lake Zurich, the Limmat River, and Zurich's city center), and neutral demographic processes subsequently shaped patterns of genetic diversity.

This work was among the first to document genetic patterns of urban colonization. However, through use of neutral microsatellites, it could only assess neutral genetic structure without considering potential adaptive differences. For example, Zurich foxes may have different selection pressures associated with anthropogenic food availability (Contesse, Hegglin, Gloor, Bontadina, & Deplazes, 2004), which could influence selection on metabolic pathways (Harris & Munshi‐South, 2017). City foxes may also experience different disease pressures than rural foxes, as seen with the zoonotic cestode *Echinococcus multilocularis* transmission cycle within Zurich city foxes (Hofer et al., 2000; Otero‐Abad, Rüegg, Hegglin, Deplazes, & Torgerson, 2017). These variable selection pressures, combined with shifts in behavior to avoid human interactions (Gloor et al., 2001; Sih et al., 2011; Soulsbury, Baker, Iossa, & Harris, 2010), could facilitate strong pressures on city foxes not present in rural conspecifics and shape genetic differentiation between the subpopulations.

In the present study, we revisited the question of how urbanization influences population divergence and evolutionary change by re‐assessing the city‐fox phenomenon in Zurich. Through examination of genome‐wide SNPs and MHC‐linked microsatellites, we addressed the following questions with a subset of the original Wandeler et al. (2003) samples:

1. Do we observe subpopulation structure between urban and rural foxes in Zurich?
2. What patterns of genetic diversity characterize urban fox colonization?
3. Can we identify evidence of selection at MHC‐linked markers or outlier SNPs associated with urban colonization?

Given the recent timing of this urban colonization and the generalist life history of red foxes, we hypothesized that fine‐scale population structure would separate adjacent rural and urban foxes. Though foxes are capable of dispersing long distances, the localized radio‐tracking data reported by Gloor (2002) suggested minimal movement across the rural–urban border. We anticipated further structure between subpopulations east and west of Lake Zurich, the Limmat River, and the Zurich city center, following the results of Wandeler et al. (2003). Regarding patterns of diversity, we predicted that urban foxes would exhibit decreased variation consistent with founder events when compared to adjacent rural populations. We likewise anticipated a smaller effective population size of urban‐dwelling foxes. Lastly, though we predicted that all marker types would exhibit decreased diversity in urban foxes, we anticipated identification of outlier SNPs associated with potentially adaptive urban phenotypes (such as carbohydrate metabolism or exploratory behavior). We additionally expected to find evidence of balancing selection at MHC Class I and II markers (which function in parasite recognition) and directional selection at MHC class III markers (which function in protein secretion). Whereas the neutral markers examined in Wandeler et al. (2003) were limited to demographic inference, the functionally linked and genomic datasets presented herein allowed for consideration of both stochastic demographic events and local adaptation as important evolutionary forces shaping genetic diversity during urban colonization.

## 2 MATERIALS AND METHODS

### 2.1 Study system

We re‐evaluated urban and rural fox samples collected in the greater Zurich area from spring 1996 to autumn 1998 (Princeton IACUC #2056A‐16). Sample selection is detailed in Wandeler et al. (2003), but briefly, we included samples from two urban environments (*U*_east_ and *U*_west_) that were separated by Lake Zurich, the Limmat River, and the dense Zurich city center (Gloor, 2002). Three rural areas (*R*_east_, *R*_west_, and *R*_north_) were selected from within a 20 km radius of Zurich's city center to represent the surrounding rural fox population. We only included individuals believed to be resident foxes based on sampling location, season, sex, and age to avoid erroneous inclusion of dispersing juveniles or males traveling through multiple territories during mating season. We found the methods described by Wandeler et al. (2003) sufficient to establish residency, especially given the relatively small territory sizes (mean = 0.28–0.40 km^2^) of foxes sampled around Zurich and the restricted movement observed between habitat types (Contesse et al., 2004; Gloor, 2002; Soulsbury et al., 2010).

Due to sample availability and quality requirements, we analyzed a subset of the 128 fox samples used in the original Wandeler et al. (2003) study. Our final samples sizes were 50 foxes (31 rural, 19 urban) for genome‐wide SNP analyses and 100 foxes (57 rural, 43 urban) for MHC‐linked microsatellite analyses (see Tables 1 and 2 for the number of foxes sampled in each sampling area).

**Table 1:** Mean diversity statistics calculated with 10,149 SNP loci for each subpopulation sampled

Sampling area	*N*	%Poly	PAS	*P*	*H_O_*	*H_E_*	π	*F*_IS_
*R*_west_	13	64.105 (0.010)	736	0.927 (0.001)	0.101 (0.001)	0.117 (0.001)	0.122 (0.001)	0.087 (0.010)
*R*_east_	12	62.184 (0.009)	571	0.927 (0.001)	0.103 (0.001)	0.117 (0.001)	0.122 (0.001)	0.076 (0.009)
*R*_north_	6	42.645 (0.007)	322	0.929 (0.001)	0.101 (0.002)	0.109 (0.001)	0.120 (0.002)	0.053 (0.007)
*U*_east_	13	58.548 (0.006)	463	0.928 (0.001)	0.112 (0.002)	0.113 (0.001)	0.118 (0.001)	0.023 (0.006)
*U*_west_	6	43.699 (0.006)	218	0.924 (0.001)	0.115 (0.002)	0.115 (0.002)	0.127 (0.002)	0.032 (0.006)

Notes

Reported metrics of diversity include % polymorphic sites (%Poly), number of private alleles (PAS), major allele frequency (*P*), observed heterozygosity (*H_O_*), expected heterozygosity (*H_E_*), nucleotide diversity (π), and inbreeding coefficient (*F*_IS_). Standard error is given in parentheses

**Table 2:** Mean diversity statistics calculated with nine MHC‐linked microsatellite loci for each subpopulation sampled

Sampling area	*N*	*N_A_*	PAS	*H_O_*	*H_E_*	*F*_IS_
*R*_west_	21	4.222 (0.969)	2	0.562 (0.082)	0.529 (0.081)	−0.078 (0.029)
*R*_east_	21	4.111 (0.964)	2	0.522 (0.084)	0.523 (0.078)	0.052 (0.091)
*R*_north_	15	3.778 (0.662)	1	0.587 (0.116)	0.496 (0.097)	−0.162 (0.039)
*U*_east_	32	3.333 (0.799)	0	0.462 (0.088)	0.465 (0.084)	0.020 (0.048)
*U*_west_	11	2.889 (0.539)	1	0.407 (0.111)	0.392 (0.109)	−0.055 (0.031)

Notes

Reported metrics of diversity include number of alleles (*N_A_*), number of private alleles (PAS), observed heterozygosity (*H_O_*), expected heterozygosity (*H_E_*), and inbreeding coefficient (*F*_IS_). Standard error is given in parentheses.

### 2.2 DNA extraction and restriction‐associated DNA sequencing

Genomic DNA was previously extracted with a standard phenol chloroform protocol (Bruford, Hanotte, Brookfield, & Burke, 1992), a QIAamp tissue extraction kit (Qiagen, Inc.), or a Chelex‐protocol (Goossens, Waits, & Taberlet, 1998), depending on tissue type (see Wandeler et al., 2003 for details). We quantified DNA with PicoGreen assays and screened for quality with 1% agarose gels, only including samples that had high molecular weight. We then standardized samples to 5 ng/μl.

To generate genome‐wide SNPs, we conducted restriction‐associated DNA sequencing (RADseq) on all samples that passed DNA quality standards, using a dual‐adapter/barcode‐ligated library preparation method as described by Ali et al. (2016). DNA was digested with restriction enzyme *sbfI*, individually barcoded with ligated biotinylated adapters, pooled, and sheared to 400 bp with a Covaris LE220. We used a streptavidin bead‐binding assay (Dynabeads M‐280, Invitrogen) to select fragments with the ligated barcode and eluted selected DNA in 55 μl low TE. Libraries were then prepared following standard recommendations for NEBNext Ultra ll DNA Library Prep Kit, where we used Agencourt AMPure XP magnetic beads for library purification and size selection. We standardized final libraries to 10 nM and used paired‐end DNA sequencing (2X150nt) on an Illumina HiSeq 2500.

We processed raw sequencing reads with a custom Perl script (sbfI_flip_trim_150821.pl, see Supporting information) which put all forward and reverse reads with a restriction enzyme cutsite into a single file. We then used *STACKS* *v1.42* (Catchen, Hohenlohe, Bassham, Amores, & Cresko, 2013) to continue data processing. We demultiplexed samples with *process_radtags*, where reads were discarded if they had more than two barcode errors or a quality score below 90% based on a sliding window assessment of sequence quality. We next filtered reads for PCR duplicates using *clone_filter* default parameters and mapped reads to the reference dog CanFam3.1 assembly (Lindblad‐Toh et al., 2005) using *STAMPY v1.0.21* (Lunter and Goodson 2011). To call genome‐wide SNPs, we used the *ref_map* pipeline with ‐m (minimum number of raw reads needed to create a stack) set to the default parameter 3. SNPs were further filtered with *populations,* where we retained only the first SNP per locus (‐‐write‐single‐snp flag) and removed loci with missing data for more than 10% of foxes (‐*r* 0.90). After SNP genotyping, we additionally removed individual foxes with missing data for >10% genotyped loci. Lastly, we filtered for statistical linkage disequilibrium (LD) in *PLINK* with ‐‐indep‐pairwise 50 5 0.3 flag. We evaluated this final, filtered SNP dataset for outliers and aberrant genotypes by implementing principal component analysis with *flashPCA* to confirm final sample selection (Abraham & Inouye, 2014).

### 2.3 MHC‐linked microsatellite genotyping

We selected twelve MHC‐linked microsatellites designed for domestic dogs (*C. familiaris*) after successful cross‐species amplification in red foxes (Debenham et al., 2005; R. Wayne, *personal communication*; Supporting information Table S1). These included microsatellites linked to MHC Class I (*n* = 2 loci), Class II (*n* = 8 loci), and Class III (*n* = 2 loci) genes. Multiplex PCRs were conducted in 10 μl reactions containing 5 μl Qiagen Multiplex PCR Master Mix (Qiagen, Inc.), 1 μl primer mix, 2.1 μl deionized water (diH2O), 0.4 μl bovine serum albumin (Roche, 10 mg/ml), and 1.5 μl template DNA (standardized to 5 ng/μl with diH2O). In each multiplex, forward primers were marked with one of four dye labels (6FAM, NED, PET, and VIC), and primer mixes consisted of 84 μl diH2O mixed with 2 μl 100 μM dye‐labeled forward primer and 2 μl 100 μM reverse primer for each primer pair included. Cycling conditions consisted of initial denaturation 95ºC for 5 min followed by 28 cycles of 95ºC for 30 s, 60ºC for 90 s, and 72ºC for 30 s and a final extension 60ºC for 30 min.

We diluted 1 μl of PCR product in 20 μl diH2O and thoroughly mixed 2 μl of diluted product with 9.5 μl of a formamide denaturing solution (1 ml ABI HiDi and 15 μl GeneScan™ 500 LIZ™ dye size standard). We shipped these diluted ready‐to‐run samples to the Biotechnology Resource Center at Cornell University Institute of Biotechnology for fragment analysis. Genotypes were assigned by visual inspection of fragment analysis peaks in *Geneious *v6.1.6 (Kearse, 2012).

We used the *R* package *Genepop *(Rousset, 2008) to evaluate LD between all pairs of loci and test for deviations from Hardy–Weinberg equilibrium (HWE) in each subpopulation across all loci and at each locus. Following Wandeler et al. (2003), we implemented the complete enumeration method for loci with fewer than five alleles and the Markov chain method for loci with more than four alleles. We then used Fisher's exact test to identify significant deviations from HWE at the subpopulation level. We determined significance levels using a modified false discovery rate (FDR) with a 5% threshold to account for multiple testing (Benjamini & Yekutieli, 2001).

### 2.4 Population structure

As traditional methods often fail to detect fine‐scale structure in urban populations (Combs, Puckett et al., 2018), we combined several related analyses (described below) to evaluate population admixture, differentiation, and possible colonization routes in Zurich foxes sampled in five areas (*i.e*., *R*_east_, *R*_west_, *R*_north_, *U*_east_, and *U*_west_) in and around Zurich. Briefly, we used (a) genetic assignment tests in *STRUCTURE *to determine the number of evolutionary clusters based on shared ancestry*, *(b) Mantel tests in the *R *package *ecodist *to evaluate spatial genetic structure within the entire sampling area, (c) principal component analysis (PCA) in *flashPCA *to identify the primary drivers of individual clustering, (d) discriminate analysis of principal components (DAPC) in the *R *package *adegenet *to explore the relationship between sampling location and population substructure, and (e) estimates of pairwise genetic differentiation (*F*_ST_) and combined private allelic richness to consider genotype differentiation and allele sharing between the groups identified in the previous analyses. Use of multiple analyses enabled us to consider population substructure in a variety of diverse yet complementary analytical frameworks. This increased confidence that observed substructure patterns resulted from true signal rather than statistical relicts of any singular method.

We first used the admixture model in *STRUCTURE 2.3.4* with the SNP dataset to assess genetic cluster assignments based on shared ancestry (Pritchard, Stephens, & Donnelly, 2000). A more traditional approach, *STRUCTURE*'s admixture model often serves as a starting point when assessing population structure. We tested a range of clusters (*K*) spanning 1–10 (or 1–2*n*, where *n* is the number of distinct sampling areas), with 10 independent runs at each *K *value. Each run had 10,000 burn‐in length and 25,000 Monte Carlo Markov Chain repetitions. We determined the optimal *K *value based on evaluation of log probabilities. Given the geographic proximity of our five sampling areas, we ran this model with sampling location given a priori using LOCPRIOR. This parameter setting aids detection of weak structure without forcing erroneous evolutionary clusters (Pritchard et al., 2000).

To explicitly consider geography without predefining sampling area, we implemented Mantel tests in the *R *package *ecodist *to measure autocorrelation between spatial and genetic variables for all Swiss foxes (Diniz‐Filho et al., 2013; Goslee & Urban, 2007). We used a Euclidean distance matrix of Swiss X‐ and Y‐coordinates for the spatial variable and a genetic distance matrix made from the SNP dataset for the genetic variable. We performed a Mantel test with 1,000 permutations and constructed a Mantel correlogram with distance classes set to 1,000 m. We chose this parameter to match the minimum home range size reported for red foxes living in human‐modified environments (Mueller, Drake, & Allen, 2018; Walton, Samelius, Odden, & Willebrand, 2017).

We next completed a PCA using the SNP dataset through *flashPCA* to explore clustering patterns in PC space without additional input information (Abraham & Inouye, 2014). Rather than using shared ancestry to assign population clusters (as in *STRUCTURE *analyses), PCA‐based approaches rely on ordination. This allowed us to consider the variables underlying emergent clusters without including prior assumptions about sampling location. To later incorporate geographic information, we used Spearman's rank correlation tests to compare PC1 with the Swiss coordinate system's X‐ and Y‐coordinates (following Combs, Puckett et al., 2018). We further explored the impact of sampling area on structure by using DAPC in the *R *package *adegenet *(Jombart, Devillard, & Balloux, 2010) to visualize differences between our five subpopulations assigned a priori. This approach differs from PCA (which describes total variance in allele frequencies) by maximizing between‐group differences while minimizing within‐group variance, thus enabling DAPC to detect subtle differences between populations. We visualized the primary discriminate functions in *adegenet *with *scatter* and constructed a simple neighbor‐joining (NJ) tree with our genetic distance matrix comparing all foxes in the *R *package *ape* (Paradis, Claude, & Strimmer, 2004; Saitou & Nei, 1987).

We lastly calculated pairwise *F*_ST_ to examine genotypic differentiation between subpopulations in *STACKS* using the *p*‐value‐corrected AMOVA *F*_ST_ method for RADseq data (Catchen et al., 2013). For MHC‐linked microsatellites, we calculated *F*_ST_ and implemented the *G* test for genotypic differentiation in *Genepop *(Rousset, 2008; Weir & Cockerham, 1984). To further estimate allele sharing between subpopulations, we calculated private allelic richness for pairwise combinations of subpopulations in *ADZE v1.0 *using the SNP dataset (Szpiech, Jakobsson, & Rosenberg, 2008).

The genetic clusters identified through these analyses (considered alongside life history, movement, and sampling data previously collected for Zurich foxes; Gloor, 2002; Soulsbury et al., 2010; Wandeler et al., 2003) provided the focal groups examined in subsequent calculations of genetic diversity statistics and tests for genetic outliers.

### 2.5 Genetic diversity statistics

We estimated the effective population size (*N_e_*) with our genome‐wide SNP dataset using the linkage disequilibrium method implemented in *NeEstimator* v2.1, although these estimates were restricted to “urban” and “rural” foxes (rather than individual subpopulations) due to small sample sizes (Do et al. 2014). We used an allele frequency threshold of 0.01 to minimize bias caused by rare alleles. Though we could not predict census sizes from *N_e_* estimates (Frankham, 1995; Luikart, Ryman, Tallmon, Schwartz, & Allendorf, 2010), we used relative values of *N_e_* alongside calculated diversity statistics to inform our understanding of Zurich fox demography.

We next calculated summary and per‐population metrics of diversity with the *populations* module in *STACKS v1.42* for RADseq data (Catchen et al., 2013) and with *GenAlEx v6.503* for MHC‐linked microsatellite data (Peakall & Smouse, 2012). These metrics included percentage of polymorphic sites (%Poly; RADseq only), number of private alleles (PAS), major allele frequency (*P*; RADseq only), observed heterozygosity (*H_O_*), expected heterozygosity (*H_E_*), number of alleles (*N_A_*; microsatellites only), nucleotide diversity (π; RADseq only), and inbreeding coefficient (*F*_IS_). We then performed nested ANOVAs using base functions in *R* to compare diversity statistics between subpopulations (where specific sampling area was nested within urban/rural habitat).

Due to sample size differences in both datasets, we used a rarefaction approach to estimate allelic richness detected in each subpopulation with increasing sample size in *ADZE v1.0* (Szpiech et al., 2008). For RADseq data, we set the maximum standardized sample size to 100 and the missing data tolerance to 25% (Szpiech et al., 2008), in order to maximize rarefied sample size (*g*) while retaining the majority of loci. For microsatellite data (where more foxes were genotyped at fewer loci), we set the maximum standardized sample size to 200 and the missing data tolerance to one, in order to retain all loci for analysis.

To explore potential effects of inbreeding, we used the SNP dataset to identify runs of homozygosity (ROH) in each subpopulation with the *R *package *detectRUNS *(Biscarini, Cozzi, Gaspa, & Marras, 2018). Following Marras et al. (2015), we used the consecutive runs method for ROH detection and required that ROHs contain a minimum of 15 SNPs, a maximum inter‐SNP distance of 1 Mb, and no heterozygous or missing genotypes. We set the minimum ROH length to 100 Kb due to the high incidence of short ROHs detected in mammals (Ceballos, Joshi, Clark, Ramsay, & Wilson, 2018; Frazer et al., 2007).

### 2.6 Identifying outlier loci

To control for relicts of demographic history, we adopted a similar multifaceted approach to identify outlier loci associated with urbanization. We performed pairwise subpopulation comparisons in *pcadapt *to detect outliers in clusters identified through previous structure analyses (Luu, Bazin, & Blum, 2017). We then performed an overall comparison of all urban and rural foxes in *GEMMA *while controlling for sampling location and relatedness (Zhou & Stephens, 2012, 2014). Given the current debate surrounding the use of RADseq data for outlier detection (Catchen et al., 2017; Lowry et al., 2016; McKinney, Larson, Seeb, & Seeb, 2017), recurrence of outliers or putative gene functions in multiple analyses increased confidence that results were true signal rather than demographic effects.

We first used the genome scan method implemented with the *R *package *pcadapt* to detect outliers in the context of population structure (Luu et al., 2017). This method outperforms other genome scan methods in the presence of admixture, population divergence, and range expansion, while also minimizing false discoveries. It first uses principal component analysis of all SNPs to ascertain genetic clusters and subsequently regresses each SNP against *K *principal components (specified by the user) to obtain a vector of *z*‐scores. Outlier loci are identified from these vectors by calculating Mahalanobis distance (*D*) test statistics, which measure the multidimensional distance of each point from the mean. We determined significance by transforming *p*‐values into *q*‐values with the *R *package *qvalue* (John D. Storey with contributions from Andrew J. Bass, Alan Dabney and David Robinson (2015). qvalue: Q‐value estimation for false discovery rate control. R package version 2.12.0. http://github.com/jdstorey/qvalue)*,* retained SNPs with *q* < 0.05, and focused attention on outlier SNPs detected in multiple analyses.

We next used the software package *GEMMA* to identify outlier loci that may be associated with urban fox colonization (Zhou & Stephens, 2012, 2014). *GEMMA *fits a univariate linear mixed model that tests each SNP for association with the specified phenotype (i.e., urban or rural, based on individual sampling location) while accounting for population stratification and designated covariates. We calculated a centered relatedness matrix from the SNP dataset using the ‐‐gk 1 flag in *GEMMA* and included sex and subpopulation as covariates. We applied a modified FDR to adjust the 5% significance threshold to correct for multiple testing (Benjamini & Yekutieli, 2001).

To consider the possible functional relevance of outliers identified through *pcadapt *and *GEMMA *analyses, we annotated outlier sites in relation to the reference dog genome (Lindblad‐Toh et al., 2005) with our in‐house python script (vonHoldt, Heppenheimer, Petrenko, Croonquist, & Rutledge, 2017). We then queried SNPs located in introns, exons, and promoters in the Ensembl, OMIM, and GeneCards databases. We additionally ran each SNP through the Ensembl Variant Effect Predictor (VEP) to annotate outliers for possible functional effects (McLaren et al., 2016).

For MHC‐linked markers, we implemented the Ewens–Watterson homozygosity test of neutrality in *PyPop* to test each locus for evidence of selection at these candidate loci (Lancaster, Single, Solberg, Nelson, & Thomson, 2007). In this test, the normalized deviate of homozygosity (*F*_ND_) compares observed homozygosity to that expected under neutral conditions. Significantly negative *F*_ND _values indicate balancing selection, as observed homozygosity is lower than expected homozygosity. This result suggests that genetic diversity is actively maintained. Alternatively, significantly positive *F*_ND_ values indicate directional selection, as observed homozygosity is higher than expected. This result suggests maintenance of a few particular alleles that may confer selective advantage. For this analysis, we considered both statistically significant results and general trends as potentially biologically relevant, given constraints of sample size.

## 3 RESULTS

### 3.1 Restriction‐associated DNA sequencing

A total of 72 foxes passed quality control screening and were prepared for RADseq, 50 of which retained enough quality reads to be included in downstream analyses. Overall, we sequenced a total of 258,360,568 paired‐end reads and retained 172,305,248 (66.7%) after removing reads without a barcode. Of these, another 5.8% of sequenced reads were removed due to poor quality with *process_radtags* in *STACKS*. Lastly, an average of 10% of reads per individual (6%–36%) were removed as PCR duplicates with *clone_filter*. Our final dataset consisted of 10,149 SNPs filtered for LD in 31 rural and 19 urban foxes. This served as our final SNP dataset in all subsequent analyses.

### 3.2 MHC‐linked microsatellite genotyping

Given lower DNA quality requirements for microsatellites, we genotyped 115 foxes at 12 MHC‐linked microsatellite loci. We discarded three markers (ABCF1_INTRO1, CFA12‐2, and CFA12‐15) due to monomorphism, leaving nine polymorphic loci for downstream analyses. We then removed individual foxes that failed to amplify three or more loci (*i.e*., missing data >30% following Heppenheimer et al., 2017; vonHoldt et al.., 2008), leading to a final dataset of 100 foxes. This dataset consisted of 57 rural and 43 urban foxes. Mean successful amplification rate for the nine polymorphic loci included in this dataset was 97.67%, with a minimum of 94.00% for C2_BF_2 and maximum of 100.00% for CFA12‐21 and CFA12‐19.

Significant LD was found in eleven pairs of MHC‐linked loci across all individuals after applying modified FDR correction (Benjamini & Yekutieli, 2001; Supporting information Table S2). As these loci derive from the same gene family, we anticipated statistical linkage at these markers due to their presumed physical proximity or functional similarity (Debenham et al., 2005). We therefore retained all loci for analysis.

No subpopulation reported significant deviation from HWE across all loci (Fisher's exact test; Supporting information Table S3). We additionally tested each locus within each subpopulation (exact HW test) and observed no significant deviations after correcting for multiple testing (modified FDR; Benjamini & Yekutieli, 2001). As a result, we used all nine loci for downstream analyses.

Rural and urban foxes reported similar numbers of successfully amplified loci (means of 8.82 and 8.74, respectively) with no significant difference between them (*χ^2^ = *0.617, *p* = 0.735; *χ^2^*‐test of independence). In total, 44 alleles were amplified across all nine loci, with a minimum of 2 alleles detected for C2_BF_1, C2_BF_2, and CFA12‐19 and a maximum of 13 alleles detected for CFA12‐17.

### 3.3 Population structure

We observed minimal differences between results using our full SNP dataset (*n* = 10,149 loci) and a subdivided dataset of intergenic sites (*n* = 5,439 loci; Supporting information Appendix S2). We therefore retained all SNPs for downstream analyses to maximize informational content.

Results from the *STRUCTURE *admixture model with LOCPRIOR conditions returned an optimal *K *of one evolutionary cluster based on the mean log probability of *K* (Supporting information Figure S1). These results suggested shared ancestry between all foxes sampled within and around Zurich and positioned all subsequent analyses to focus on finer‐scale substructure.

Within this evolutionary cluster, the overall Mantel test and Mantel correlogram supported patterns of spatial genetic structure (Supporting information Figure S2). Foxes sampled within 3,000–4,000 m of one another exhibited positive and statistically significant spatial autocorrelation, with foxes sampled more than 6,000 m apart exhibiting negative autocorrelation. The highest correlation was observed between pairs of foxes 0–1,000 m (*r = *0.207, *p = *0.002), with the overall Mantel test suggesting moderate correlation across the full range of sampling distances (*r = *0.288, *p = *0.001).

**Figure 2:**
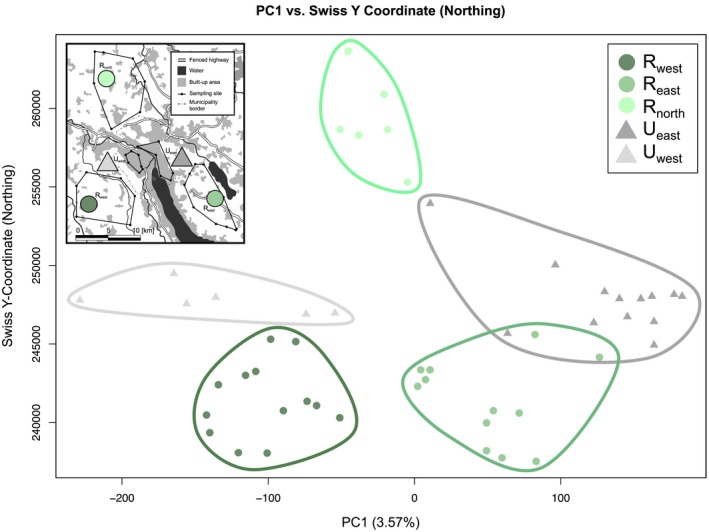
Principal components calculated for 50 foxes across all 10,149 SNPs. When plotted against the Swiss Y‐coordinate (northing), PC1 recapitulates the geographic sampling area (inset; adapted from Wandeler et al. 2003), thus mirroring the Swiss X‐coordinate (easting).)

Geographic substructure was further supported by the principal components calculated for the SNP dataset in *flashPCA*. Foxes sampled within each subpopulation clustered together, with overlap observed between abutting populations (Supporting information Figure S3). Along PC1 (3.57% of variance), the urban populations are farthest apart, with populations west of Lake Zurich, the Limmat River, and Zurich's city center (*U*_west_ + *R*_west_) clustered to the left and those east of the three landmarks (*U*_east_ + *R*_east_) clustered to the right. The rural population sampled north of the city (*R_north_*) fell in the center. The component explaining most variance (PC1) was significantly correlated with the Swiss X‐coordinate (easting) in the Spearman's rank correlation test (*ρ* = 0.689, *p* < 0.001), with no association detected between PC1 and the Swiss Y‐coordinate (northing; *ρ* = 0.173, *p = *0.230). Plotting PC1 against the Swiss Y‐coordinate recapitulated the geographic sampling area of Zurich foxes (Figure 2), suggesting a prominent role of geography in underlying population substructure.

**Figure 3:**
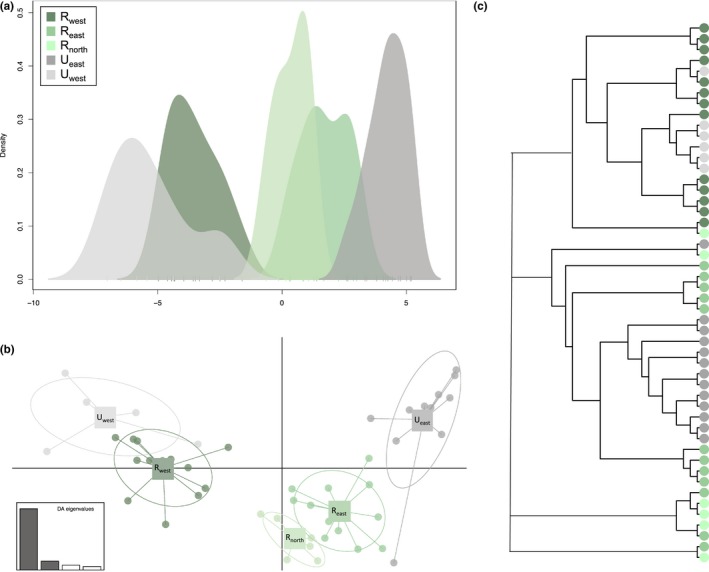
Discriminate analysis of principal components (DAPC) revealed (a) considerable overlap between the five sampling locations, with five distinct groups evident and (b) a divide between sampling locations east (*R*_east_ and *U*_east_) and west (*R*_west _and *U*_west_) of Lake Zurich and the Limmat River, with *R*_north _in the middle. (c) East–west subdivision also emerged in the major branches of the NJ tree, where each node represents an individual fox colored by sampling location

To further explore this relationship, we implemented DAPC with 10 retained PCs and a priori subpopulation assignments to *R*_east_, *R*_west_, *R*_north,_ *U*_east_, and *U*_west_ (Figure 3). The first discriminate function showed considerable overlap between sampling areas, with all five clusters evident nonetheless (Figure 3a). Retention of two discriminate functions (Figure 3b) closely matched PCA results, with less overlap between subpopulations and tighter clustering within subpopulations. The divide between sampling areas east (*R*_east_ and *U*_east_) and west (*R*_west _and *U*_west_) of Lake Zurich, the Limmat River, and Zurich's city center further emerged in this analysis, with large branches of the NJ tree broadly corresponding to this geographic subdivision across the lake and river (Figure 3c). Once again, *R*_north_ was situated between the east–west clusters in the DAPC plots, with assignment to different branches of the NJ tree.

We next considered differentiation between the five clusters observed in our PCA and DAPC plots. At MHC‐linked microsatellite loci, significant differences were reported for most pairs of populations (*G* test) after correcting for multiple testing, with the exceptions of *R*_north_ + *R*_east_, *R*_north_ + *R*_west_, and *R*_east_ + *U*_east _(Supporting information Table S4). This result suggested little immunogenetic differentiation between rural populations and adjacent rural and urban populations on the east side of the Limmat River.

Further calculations of pairwise *F*_ST_ using SNP and MHC‐linked loci revealed similar patterns to those reported by Wandeler et al. (2003). The largest *F*_ST_ values (SNP = 0.0135, MHC‐linked microsatellite = 0.1153) occurred between the two urban subpopulations, *U*_east_ + *U*_west _(Supporting information Tables S4 and S5). We calculated *F*_ST_ of a similar magnitude between urban and rural subpopulations separated by Lake Zurich, the Limmat River, and Zurich's city center, with the lowest values reported between rural subpopulations and adjacent rural–urban subpopulations. As such, private allelic richness for pairwise combinations of subpopulations was highest for adjacent rural–urban subpopulations (*R*_west_ + *U*_west_ = 0.0360; *R*_east_ + *U*_east_ = 0.0324) and lowest for two pairings separated by the center city barriers (*U*_west_ + *U*_east_ = 0.0189; *R*_west_ + *U*_east_ = 0.0181; Supporting information Table S6).

Taken together, these results (considered alongside radio‐tracking and home range data; Gloor, 2002; Gloor et al., 2001; Soulsbury et al., 2010) supported analysis of five subpopulations in subsequent calculations of genetic diversity and outlier detection tests. Though comprising one evolutionary lineage, foxes sampled within and around Zurich exhibited geographic substructure confirmed by diverse yet complementary analyses.

### 3.4 Genetic diversity statistics

Estimates for *N_e_* using the LD method were 41.0 for urban (*U*_east_ + *U*_west_) foxes and 5,915.5 for rural (*R*_east_ + *R*_west_ + *R*_north_) foxes. Though N_e_ estimates should not be mistaken for projected census sizes, they do suggest far greater numbers of foxes outside of the city limits.

Genetic diversity statistics for the SNP dataset are reported in Table 1. Both urban subpopulations exhibited reduced allelic diversity when compared to rural subpopulations with similar sample sizes, as measured by the number of private alleles. To compare allelic diversity across all areas, we used a rarefaction approach implemented in *ADZE v1.0* to calculate allelic richness at standardized sample sizes (Szpiech et al., 2008). Here, *U*_east_ exhibited the lowest allelic richness of all subpopulations, with the remaining four subpopulations clustered together (Supporting information Figure S4A).

Heterozygosity deviated from this trend of reduced urban diversity (Table 1). Though expected heterozygosity was similar in all five subpopulations (*H_O_* range = 0.101–0.115), urban foxes reported higher observed heterozygosity and lower inbreeding coefficients than the rural subpopulations. For heterozygosity, nested ANOVA reported a significant effect of habitat (*F = *72.754, *p* < 0.001), with an insignificant effect of subpopulation (*F* = 1.198, *p* = 0.309). For inbreeding coefficients, both habitat (*F = *378.09, *p < *0.001) and subpopulation (*F* = 34.54, *p < *0.001) were statistically significant.

At MHC‐linked microsatellites, urban subpopulations reported lower values of observed heterozygosity than rural foxes (Table 2). At these loci, nested ANOVA reported a significant effect of habitat (*F* = 7.820, *p* = 0.006), with an insignificant effect of individual sampling area (*F = *0.777, *p* = 0.510). Mean number of alleles was similarly reduced in urban foxes, although neither habitat nor sampling area had statistically significant effects (*p* > 0.05). Across standardized sample sizes, rarefaction of allelic richness showed fewer alleles in urban foxes when compared to rural foxes (Supporting information Figure S4B).

**Figure 4:**
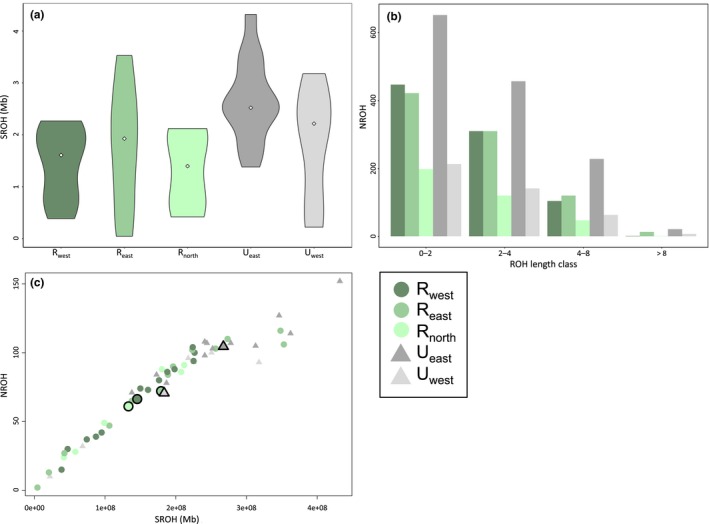
Plots displaying (a) the sum of ROH (SROH) in Mb calculated per individual per subpopulation (median values indicated by white diamonds; nested ANOVA reported significant effect of urban vs. rural habitat on SROH; *F = *9.843, *p = *0.003), (b) the total number of ROHs (NROH) observed at each length class (0–2; 2–4; 4–8; >8 Mbs; nested ANOVA again reported significant effect of habitat on NROH; *F = *7.91, *p = *0.007) in each subpopulation (note sample size sensitivity of this metric), and (c) SROH vs. NROH per individual fox and averaged for each subpopulation (large points with black outlines; *r^2^* = 0.918)

Analysis of ROH by subpopulation revealed similar patterns of decreased diversity in urban foxes (Figure 4). Median values for the sum of all ROH detected in each individual were higher in urban foxes, with the *U*_east_ distribution notably shifted upwards (Figure 4a). This trend was statistically significant, as nested ANOVA of the sum of ROH reported a significant effect of habitat (*F = *9.843, *p = *0.003), with specific sampling area insignificant (*F = *1.546, *p = *0.216).

A similar pattern emerged in total number of ROH observed per length class (Figure 4b). It is important to note that this metric is highly influenced by sample size, as the total number of ROH detected will positively increase with the number of individuals sampled. We therefore restricted qualitative comparison to subpopulations with equal sample sizes (*R*_east_, *R*_west,_ *U*_east_ with 12–13 foxes each; *R*_north_, *U*_west_ with six foxes each) and observed that both urban subpopulations consistently possessed more ROH than rural foxes, with *U*_east_ exhibiting the global maximum in each length class. As with the sum of ROH, nested ANOVA of the number of ROH reported a significant effect of habitat (*F = *7.91, *p = *0.007) with subpopulation effect insignificant (*F = *1.690, *p = *0.1826).

We lastly observed a strong positive correlation between the sum of ROH and the number of ROH calculated per fox (*r^2^* = 0.918), with urban foxes (particularly *U*_east_) more densely concentrated in the upper right quadrant of the plot than rural foxes (Figure 4c). This trend is reflected by mean values calculated for each subpopulation. As we expect newly colonized populations to exhibit more ROHs than larger source populations, these analyses aided our understanding of fox colonization history in and around Zurich.

### 3.5 Identifying outlier loci

Following our population structure results, we analyzed foxes east (*R*_east_ + *U*_east_) and west (*R*_west_ + *U*_west_) of Lake Zurich, the Limmat River, and Zurich's city center separately in *pcadapt*. We excluded *R*_north_ from this analysis due to its intermediate position between *R*_east _and *R*_west _in the PCA and DAPC analyses. For both runs of *pcadapt*, we regressed the SNP dataset (*n* = 10,149 loci) against the first two PCs, since *K = *1–2 captured the relevant population structure in this and previous (DAPC) analyses. Using a significance threshold of *q* < 0.05, we detected 514 outliers in east subpopulations and 508 outliers in west subpopulations, with 42 loci appearing as outliers in both analyses. These overlapping SNPs included 17 annotated as intergenic, 27 as intronic, three as exonic, and two near promoters (note that some SNPs have multiple annotations). We queried genic outliers significant in both analyses (Supporting information Table S7) in the Ensembl, OMIM, and GeneCards databases, and observed numerous functions related to energy metabolism, drug tolerance, and immune processes. Ensembl VEP annotations included “low impact” (i.e., minimal effect on protein), “moderate impact” (i.e., may alter protein effectiveness), and “modifier” (i.e., no known effects on protein).

We next examined SNPs associated with urban versus rural habitats in the full fox dataset (*n* = 50 individuals), using *GEMMA* to fit a univariate linear mixed model with sex and sampling area as covariates. We further controlled for population structure by including a relatedness matrix of all foxes sampled. We analyzed all 10,149 SNPs and adjusted our likelihood ratio test (*lrt*) significance threshold using the modified FDR (adjusted *p = *0.005). This analysis produced 91 SNPs significantly associated with the urban phenotype, with 48 annotated as intergenic, 42 as intronic, 11 as exonic, and three near promoters (again, note that some SNPs have multiple annotations). Although no SNPs overlapped with outliers identified in *pcadapt*, possible gene functions once again related predominantly to immunity and metabolism (among other cellular processes), and VEP analysis included “low impact,” “moderate impact,” and “modifier” annotations.

To identify outlier loci in the MHC‐linked microsatellite dataset, we implemented the Ewens–Watterson homozygosity test of neutrality in *PyPop *(Supporting information Figure S5; Table S8). Each subpopulation had one or two loci with significantly negative *F*_ND_ values (after correcting for multiple testing), which suggested the presence of balancing selection at loci linked to MHC Class I and II genes. Though remaining loci were not significantly positive or negative, we considered trends as potentially biologically relevant, as small sample sizes may have limited our ability to detect statistically significant relationships (Krausman, 2017). As such, we noted that the majority of *F*_ND _values were negative at MHC Class I and II markers. Two exceptions linked to MHC Class III genes (C2_BF_1 and C2_BF_2) exhibited positive F_ND_ values in subpopulations polymorphic at those markers, which may suggest positive selection.

## 4 DISCUSSION

In the present study, we revisited the city‐fox phenomenon in Zurich, Switzerland, to examine the genetic effects of urban colonization and the evolutionary processes that shaped them. Through concurrent analysis of genome‐wide SNPs and MHC‐linked microsatellites, we addressed the following questions: (1) Do we observe subpopulation structure between urban and rural foxes in Zurich? (2) What patterns of genetic diversity characterize urban fox colonization? (3) Can we identify evidence of selection at MHC‐linked markers or outlier SNPs associated with urban colonization?

Considered together, our results suggested the presence of one evolutionary cluster subdivided into smaller groups clustered by geographic sampling area. We observed significant differentiation across natural (i.e., Lake Zurich and the Limmat River) and anthropogenic (i.e., urban infrastructure separating adjacent rural and urban foxes) barriers that were consistent across multiple data‐types and complementary analytical frameworks. These results matched previous radio‐tracking data that suggested smaller home range sizes and localized movement of foxes inhabiting rural and urban areas around Zurich (Gloor, 2002). They further matched results reported by similar studies examining the genetic effects of natural (*C. lupus; *Fabbri et al., 2007) and urban colonization (*C. latrans; *DeCandia et al., 2019), species reintroduction (*Etheostoma fonticola*; Olsen et al., 2016), and habitat fragmentation (*Propithecus perrieri*; Salmona et al., 2015).

Regarding genetic diversity, this study builds upon the results reported by Wandeler et al. (2003) by showing a more complicated pattern at functionally linked and genome‐wide loci. Although we observed reduced diversity at both marker types, this pattern was more nuanced across different diversity metrics. For example, genome‐wide allelic richness decreased and runs of homozygosity increased amid relatively high levels of observed heterozygosity in urban foxes. This pattern is characteristic of recently bottlenecked populations. As rare alleles typically decline faster than genome‐wide heterozygosity, there often exists a temporal lag between these diversity metrics before populations reach a new equilibrium (Cornuet & Luikart, 1996). Given the recent nature of this colonization event and subtleties of within‐population clustering, we anticipate no long‐term negative effects of these declines.

We further detected outlier loci that may provide preliminary evidence of urban adaptation during colonization. Although we observed no overlap between outlier SNPs using different outlier detection methods, we did observe considerable overlap in possible gene functions. Recurring annotations were related to metabolism (e.g., *SORT1 *and *NNMT*; Shi & Kandror, 2005; Kraus et al., 2014), drug tolerance (e.g., *NNMT*; Aksoy, Szumlanski, & Weinshilboum, 1994), and immune processes (e.g., *CD22 *and *ATG16L1*; O'Keefe, Williams, Davies, & Neuberger, 1996; Chu, 2016). Two genes even had behavioral annotations relevant to urban colonization, such as exploration (*NAV2*; Peeters et al., 2004), locomotor activity, circadian rhythms, and fear conditioning (*RAI1*; Walz et al., 2004; Girirajan et al., 2008). Across taxa, outlier SNPs have been identified with similar gene functions, including lipid and carbohydrate metabolism (*P. leucopus; *Harris & Munshi‐South, 2017), harm avoidance behavior (*Turdus merula*; Mueller et al., 2013), toxicant exposure (*Fundulus heteroclitus*; Reid et al., 2016), and immune processes (*J. hyemalis; *Whittaker et al., 2012; *L. rufus*; Serieys et al., 2015).

These results should be interpreted with caution, due to the small sample size, minimal overlap of specific outlier sites, and ongoing debate on the use of RADseq data for detecting genomic outliers (Catchen et al., 2017; Lowry et al., 2016; McKinney et al., 2017). However, identification of colonization‐relevant functions despite these limitations provides motivation for later studies examining contemporary samples with larger‐scale datasets that can more exhaustively scan the genome. Similarly, though the majority of the MHC‐linked loci did not statistically deviate from neutrality, overall patterns may still be informative. For example, we detected possible balancing selection at MHC Class I and II loci, compared to possible directional selection acting on MHC Class III loci. Interpretations must currently remain conservative, as it is difficult to parse contemporary from historical signatures of selection in recently bottlenecked populations (Gilroy, Phillips, Richardson, & Oosterhout, 2017).

Through examination of genome‐wide SNPs and MHC‐linked microsatellites, this study expanded upon the results reported by Wandeler et al. (2003) to provide a more comprehensive understanding of the genetic effects of urban fox colonization and the evolutionary processes that shaped them. We reported patterns of genetic variation consistent with founder events and increased differentiation consistent with natural and anthropogenic barriers to dispersal. We additionally identified outlier loci with putative gene functions related to urban‐associated processes, such as energy metabolism, behavior, and immunity. In addition to system‐specific information gained, this study contributes toward our overall understanding of the genetics of urban evolution, an exciting frontier within urban evolutionary biology (Johnson & Munshi‐South, 2017; Santangelo et al., 2018). It further emphasizes the value of combining datasets to parse the roles of stochastic and adaptive processes underlying evolutionary change in an increasingly urban world.

## CONFLICT OF INTEREST

None Declared.

## AUTHOR CONTRIBUTIONS

ALD, KEB, and BMvH designed the study; GC, PW, and CD contributed samples; ALD, KEB, and CC conducted the laboratory work; ALD and KEB processed the raw data; ALD analyzed the data; ALD, KEB, EH, and BMvH provided analytical assistance; ALD, KEB, and BMvH prepared the manuscript; all authors contributed to and approved the final manuscript.

## Data Availability

RADseq clone filtered and demultiplexed fastq files and CanFam3.1 mapped bam files are available through the NCBI Sequence Read Archive (SRA PRJNA510648). MHC‐linked microsatellite genotypes are available through the Dryad Digital Repository: https://doi.org/10.5061/dryad.17r39p2.
