# Supplementary material for: Urban colonization through multiple genetic lenses: The city‐fox phenomenon revisited
Source: Ecol Evol. 2019 Jan 31;9(4):2046–60. doi: 10.1002/ece3.4898 (PMC6392345; doi:10.1002/ece3.4898)
Supplement: Supplementary file 1 [file ECE3-9-2046-s001.docx]

**Appendix 1. Supplementary Tables and Figures**

**Table S1**. Primer names and sequences for 12 major histocompatibility complex (MHC) linked microsatellite loci used to survey immunogenetic variation in Swiss foxes.

| **Primer** | **MHC Class** | **Forward primer (5' --> 3')** | **Reverse primer (5' --> 3')** | **SOURCE** |
| --- | --- | --- | --- | --- |
| ABCF1_INTRO1 | I | TGTGAGGGGTGCCTGGCTGT | CCACCCCATCCACCAACCGC | R. Wayne 2015, pers. comm. |
| GABBR1_DOWN1 | I | CAGGCTCCCAGGAGGCAAGGA | GTGTGGTCAGGACCCCCGGA | R. Wayne 2015, pers. comm. |
| CFA12-2 | II | TTCTCCAGTTCCATCTACGTCA | AATGTCCACAATAGCCTAACCG | Debenham et al. 2005 |
| CFA12-6 | II | CATTCTCAGGTGTGCTC | CCTCCTTGTAACTTGGGGGT | Debenham et al. 2005 |
| CFA12-15 | II | ACCCATCCCTCTACTCTCTTCC | TGATGAACTGATCAAGATGTGG | Debenham et al. 2005 |
| CFA12-17 | II | CCCTGAGCACCTACTTTGGA | TGCATGTGGGTAGCTATGTAGG | Debenham et al. 2005 |
| CFA12-19 | II | ACCAGAGCTTTGGGTAGAACA | GCAGCTAGCTCCCCTTCC | Debenham et al. 2005 |
| CFA12-20 | II | CACTCTTTGTTCTCGCTTTGG | GGGGGTTTTCCTACCATCAT | Debenham et al. 2005 |
| CFA12-21 | II | TGGAATGATTACACAATCGAGC | AATGTGTTCAGGGTAGACCTCG | Debenham et al. 2005 |
| DRB1_INTRO1 | II | GGGTGGTCTGAATTACCTGGGTGC | TCCAGTCGAAACTGCCCTCTCC | R. Wayne 2015, pers. comm. |
| C2_BF_1 | III | GCCCCAATCTCTTGCAGGTTTATGT | ACTGGAGGCTGAGCTGAGAGGA | R. Wayne 2015, pers. comm. |
| C2_BF_2 | III | GGGGACATAGGAGTGAGATATGGGG | CCTGGGCATGGGACCACCAG | R. Wayne 2015, pers. comm. |

**Table S2.** P-values reported for linkage disequilibrium tests implemented with the *R* package *Genepop* (Rousset 2008), with significance levels determined by the modified FDR method described by Benjamini & Yekutieli (2001).

|  | C2_BF_2 | CFA12-21 | CFA12-19 | CFA12-20 | DRB1_INTRO2 | CFA12-6 | GABBR1_DOWN1 | CFA12-17 |
| --- | --- | --- | --- | --- | --- | --- | --- | --- |
| C2_BF_1 | 0.975 | 0.455 | 0.525 | 0.274 | 0.245 | 0.522 | 0.330 | 0.359 |
| C2_BF_2 | . | 0.019 | 0.006* | 0.184 | 0.611 | 0.471 | 0.241 | 0.649 |
| CFA12-21 | . | . | <0.001* | 0.001* | 0.005* | <0.001* | 0.028 | <0.001* |
| CFA12-19 | . | . | . | 0.561 | 0.243 | 0.004* | 0.338 | <0.001* |
| CFA12-20 | . | . | . | . | 0.197 | 0.009* | 0.558 | 0.021 |
| DRB1_INTRO2 | . | . | . | . | . | <0.001* | 0.639 | 0.106 |
| CFA12-6 | . | . | . | . | . | . | 0.365 | <0.001* |
| GABBR1_DOWN1 | . | . | . | . | . | . | . | 0.726 |
| * p-value below the modified FDR corrected threshold (p < 0.012) | | | | |  |  |  |  |

**Table S3.** Summary statistics are reported for each MHC-linked locus in (A) each rural subpopulation and (B) each urban subpopulation. Reported metrics of diversity include: observed heterozygosity (H_O_), expected heterozygosity (H_E_), number of alleles (N_A_), and inbreeding coefficient (F_IS_). P-values testing for significant deviations from Hardy-Weinberg equilibrium are also given (Fisher’s exact test).

| **A.** | Sampling areas (sample size) | | | |  |  |  |  |  |  |  |  |  |  | |  |  |
| --- | --- | --- | --- | --- | --- | --- | --- | --- | --- | --- | --- | --- | --- | --- | --- | --- | --- |
|  |  |  |  |  |  |  |  |  |  |  |  |  |  |  | |  |  |
|  |  |  |  |  |  |  |  |  |  |  |  |  |  |  | |  |  |
|  | R_west_ (n=21) | |  |  |  | R_east_ (n=21) | |  |  |  | R_north_ (n=15) | | | |  | |  |
|  |  |  |  |  |  |  |  |  |  |  |  |  |  |  | |  |  |
|  |  |  |  |  |  |  |  |  |  |  |  |  |  |  | |  |  |
| Locus | H_O_ | H_E_ | N_A_ | F_IS_ |  | H_O_ | H_E_ | N_A_ | F_IS_ |  | H_O_ | H_E_ | N_A_ | F_IS_ | |  |  |
|  |  |  |  |  |  |  |  |  |  |  |  |  |  |  | |  |  |
| C2_BF_1 | 0.200 | 0.180 | 2 | -0.111 |  | 0.095 | 0.172 | 2 | 0.447 |  | 0.077 | 0.074 | 2 | -0.040 | |  |  |
| C2_BF_2 | 0.200 | 0.180 | 2 | -0.111 |  | 0.095 | 0.172 | 2 | 0.447 |  | 0.077 | 0.074 | 2 | -0.040 | |  |  |
| CFA12-21 | 0.619 | 0.706 | 5 | 0.124 |  | 0.714 | 0.740 | 5 | 0.035 |  | 0.933 | 0.744 | 5 | -0.254 | |  |  |
| CFA12-19 | 0.476 | 0.408 | 2 | -0.167 |  | 0.524 | 0.500 | 2 | -0.048 |  | 0.533 | 0.391 | 2 | -0.364 | |  |  |
| CFA12-20 | 0.524 | 0.459 | 2 | -0.141 |  | 0.571 | 0.499 | 2 | -0.145 |  | 0.533 | 0.487 | 3 | -0.096 | |  |  |
| DRB1_INTRO2 | 0.667 | 0.602 | 5 | -0.107 |  | 0.619 | 0.680 | 5 | 0.090 |  | 0.933 | 0.756 | 5 | -0.235 | |  |  |
| CFA12-6 | 0.667 | 0.616 | 5 | -0.083 |  | 0.667 | 0.473 | 4 | -0.410 |  | 0.462 | 0.379 | 3 | -0.219 | |  |  |
| GABBR1_DOWN1 | 0.750 | 0.738 | 4 | -0.017 |  | 0.667 | 0.626 | 4 | -0.065 |  | 0.733 | 0.704 | 4 | -0.041 | |  |  |
| CFA12-17 | 0.952 | 0.874 | 11 | -0.089 |  | 0.750 | 0.848 | 11 | 0.115 |  | 1.000 | 0.853 | 8 | -0.172 | |  |  |
|  |  |  |  |  |  |  |  |  |  |  |  |  |  |  | |  |  |
|  |  |  |  |  |  |  |  |  |  |  |  |  |  |  | |  |  |
| Mean | 0.562 | 0.529 | 4.222 | -0.078 |  | 0.522 | 0.523 | 4.111 | 0.052 |  | 0.587 | 0.496 | 3.778 | -0.162 | |  |  |
| ± SD | 0.082 | 0.081 | 0.969 | 0.029 |  | 0.084 | 0.078 | 0.964 | 0.091 |  | 0.116 | 0.097 | 0.662 | 0.038 | |  |  |
| P (Fisher's exact test) | 0.730 |  |  |  |  | 0.148 |  |  |  |  | 0.970 |  |  |  | |  |  |
|  |  |  |  |  |  |  |  |  |  |  |  |  |  |  | |  |  |

| **B.** | Sampling areas (sample size) | | | | | | | |  | |  | |  | |  | |  | |  |
| --- | --- | --- | --- | --- | --- | --- | --- | --- | --- | --- | --- | --- | --- | --- | --- | --- | --- | --- | --- |
|  |  | |  | |  | |  | |  | |  | |  | |  | |  | |  |
|  |  | |  | |  | |  | |  | |  | |  | |  | |  | |  |
|  | U_east_ (n=32) | | | |  | |  | |  | | U_west_ (n=11) | | | |  | |  | |  |
|  |  | |  | |  | |  | |  | |  | |  | |  | |  | |  |
|  |  | |  | |  | |  | |  | |  | |  | |  | |  | |  |
| Locus | | H_O_ | | H_E_ | | N_A_ | | F_IS_ | |  | | H_O_ | | H_E_ | | N_A_ | | F_IS_ | |
|  | |  | |  | |  | |  | |  | |  | |  | |  | |  | |
| C2_BF_1 | | 0.000 | | 0.000 | | 1 | | na | |  | | 0.000 | | 0.000 | | 1 | | na | |
| C2_BF_2 | | 0.161 | | 0.200 | | 2 | | 0.195 | |  | | 0.000 | | 0.000 | | 1 | | na | |
| CFA12-21 | | 0.781 | | 0.727 | | 4 | | -0.075 | |  | | 0.636 | | 0.640 | | 3 | | 0.006 | |
| CFA12-19 | | 0.375 | | 0.451 | | 2 | | 0.169 | |  | | 0.182 | | 0.165 | | 2 | | -0.100 | |
| CFA12-20 | | 0.419 | | 0.500 | | 2 | | 0.161 | |  | | 0.091 | | 0.087 | | 2 | | -0.048 | |
| DRB1_INTRO2 | | 0.688 | | 0.651 | | 4 | | -0.056 | |  | | 0.700 | | 0.635 | | 4 | | -0.102 | |
| CFA12-6 | | 0.548 | | 0.467 | | 2 | | -0.175 | |  | | 0.556 | | 0.475 | | 3 | | -0.169 | |
| GABBR1_DOWN1 | | 0.438 | | 0.392 | | 4 | | -0.116 | |  | | 0.800 | | 0.735 | | 4 | | -0.088 | |
| CFA12-17 | | 0.750 | | 0.796 | | 9 | | 0.058 | |  | | 0.700 | | 0.790 | | 6 | | 0.114 | |
|  | |  | |  | |  | |  | |  | |  | |  | |  | |  | |
|  | |  | |  | |  | |  | |  | |  | |  | |  | |  | |
| Mean | | 0.462 | | 0.465 | | 3.333 | | 0.020 | |  | | 0.407 | | 0.392 | | 2.889 | | -0.055 | |
| ± SD | | 0.088 | | 0.084 | | 0.799 | | 0.048 | |  | | 0.111 | | 0.109 | | 0.539 | | 0.031 | |
| P (Fisher's exact test) | | 0.436 | |  | |  | |  | |  | | 0.343 | |  | |  | |  | |
|  |  | |  | |  | |  | |  | |  | |  | |  | |  | |  |

**Figure S1.** *STRUCTURE* results returned by the admixture LOCPRIOR models run with 10,149 SNPs (Pritchard et al. 2000). Examination of the mean log probability of *K* supported the presence of one genetic cluster.

**Figure S2.** The Mantel correlogram showing patterns of spatial genetic structure between pairs of foxes sampled within distance classes of 1000m (n = 50 foxes genotyped at 10,149 SNP loci). Filled circles represent statistically significant (*p* < 0.05) correlations.

**Figure S3.** Principal components calculated for 50 foxes across all 10,149 SNPs. PC1 plotted against PC2 shows that foxes sampled within the same subpopulation cluster together, with overlap observed between abutting rural and urban subpopulations.

**Table S4.** Pairwise F_ST_ values calculated with nine MHC-linked microsatellite loci between each pair of subpopulations in the *R* package *Genepop* (Rousset 2008). P-values for the *G* test of genetic differentiation are given in parentheses. Significance levels (*p* < 0.0171) were determined by a modified FDR method (Benjamini & Yekutieli 2001).

| **F_ST_** | R_east_ | R_north_ | U_east_ | U_west_ |
| --- | --- | --- | --- | --- |
| R_west_ | 0.0394 (0.0101) | 0.0129 (0.2526) | 0.0522 (<0.0001) | 0.0220 (0.0129) |
| R_east_ | . | 0.0231 (0.0887) | 0.0127 (0.0283) | 0.1148 (<0.0001) |
| R_north_ | . | . | 0.0525 (<0.0007) | 0.0363 (0.0045) |
| U_east_ | . | . | . | 0.1153 (<0.0001) |

**Table S5.** Pairwise F_ST_ values calculated with 10,149 SNP loci between each pair of subpopulations with the p-value corrected AMOVA F_ST_ method implemented in the *STACKS populations* module (Catchen et al. 2013). Standard error is given in parentheses.

| **F_ST_** | R_east_ | R_north_ | U_east_ | U_west_ |
| --- | --- | --- | --- | --- |
| R_west_ | 0.0057 (0.0003) | 0.0058 (0.0004) | 0.0109 (0.0005) | 0.0070 (0.0005) |
| R_east_ | . | 0.0056 (0.0004) | 0.0054 (0.0003) | 0.0099 (0.0006) |
| R_north_ | . | . | 0.0084 (0.0005) | 0.0078 (0.0006) |
| U_east_ | . | . | . | 0.0135 (0.0007) |

**Table S6.** Private allelic richness calculated for pairwise combinations of subpopulations in *ADZE* (Szpiech et al. 2008) with the 10,149 SNP loci. Standard error is given in parentheses.

| **Private Allele Sharing** | R_east_ | R_north_ | U_east_ | U_west_ |
| --- | --- | --- | --- | --- |
| R_west_ | 0.0230 (0.0009) | 0.0235 (0.0011) | 0.0181 (0.0007) | 0.0360 (0.0013) |
| R_east_ | . | 0.0249 (0.0011) | 0.0324 (0.0012) | 0.0218 (0.0010) |
| R_north_ | . | . | 0.0214 (0.0010) | 0.0239 (0.0012) |
| U_east_ | . | . | . | 0.0189 (0.0009) |

**Figure S4.** Rarefaction curves for the number of alleles detected with increasing sample size in each subpopulation of Swiss foxes genotyped at (A) 10,149 SNP loci and (B) nine MHC-linked microsatellite loci. Maximum *g* was limited by tolerance requirements and within-group sample sizes.

**Table S7.** Genic SNPs identified as outliers (*q* < 0.05) in both *pcadapt* (Luu et al. 2017) analyses of rural vs. urban foxes, and genic SNPs significantly associated with the urban phenotype in regression analyses implemented in *GEMMA* (Zhou & Stephens 2012, 2014; modified FDR adjusted *p*<0.005). Chromosome and base pair position, ensembl identifier, gene name, major/minor allele, analysis, ensembl variant effect predictor annotation, and minor allele frequency in each of the five subpopulations are given for each SNP.

|  |  |  |  |  |  | **Minor Allele Frequency** | | | | |
| --- | --- | --- | --- | --- | --- | --- | --- | --- | --- | --- |
| **chr.position** | **ensembl ID** | **Gene ID** | **Alleles** | **Analysis** | **VEP** | **Rwest** | **Reast** | **Rnorth** | **Ueast** | **Uwest** |
| chr1.49037406 | ENSCAFG00000000732 | TCP1 | G/C | *pcadapt* | modifier | 0.136 | 0.000 | 0.083 | 0.115 | 0.083 |
| chr1.49037406 | ENSCAFG00000031434 | MRPL18 | G/C | *pcadapt* | modifier | 0.136 | 0.000 | 0.083 | 0.115 | 0.083 |
| chr1.66773914 | ENSCAFG00000001101 | PTPRK | A/G | *pcadapt* | modifier | 0.042 | 0.250 | 0.000 | 0.077 | 0.300 |
| chr1.89373731 | ENSCAFG00000001957 | DOCK8 | C/G | *pcadapt* | modifier | 0.125 | 0.182 | 0.000 | 0.154 | 0.400 |
| chr1.117269241 | ENSCAFG00000007036 | CD22 | A/G | *pcadapt* | moderate | 0.136 | 0.045 | 0.000 | 0.115 | 0.000 |
| chr2.22647071 | ENSCAFG00000004728 | FRMD4A | T/C | *pcadapt* | modifier | 0.346 | 0.278 | 0.250 | 0.333 | 0.100 |
| chr2.73724565 | ENSCAFG00000012551 | UBXN11 | A/G | *pcadapt* | moderate/modifier | 0.292 | 0.350 | 0.083 | 0.385 | 0.100 |
| chr4.20595814 | ENSCAFG00000013957 | TSPAN15 | T/G | *pcadapt* | modifier | 0.200 | 0.000 | 0.000 | 0.154 | 0.200 |
| chr5.5925688 | ENSCAFG00000032412 | FLI1 | T/C | *pcadapt* | modifier | 0.182 | 0.273 | 0.200 | 0.125 | 0.000 |
| chr5.16087836 | ENSCAFG00000012923 | DSCAML1 | G/C | *pcadapt* | modifier | 0.167 | 0.455 | 0.167 | 0.318 | 0.000 |
| chr5.19042850 | ENSCAFG00000030573 | NNMT | T/C | *pcadapt* | low | 0.227 | 0.000 | 0.250 | 0.231 | 0.000 |
| chr5.56240812 | ENSCAFG00000019342 | AGRN | T/C | *pcadapt* | modifier | 0.318 | 0.042 | 0.083 | 0.125 | 0.100 |
| chr5.56240812 | ENSCAFG00000019348 | ISG15 | T/C | *pcadapt* | modifier | 0.318 | 0.042 | 0.083 | 0.125 | 0.100 |
| chr6.11025547 | ENSCAFG00000015351 | BAIAP2L1 | A/G | *pcadapt* | modifier | 0.100 | 0.042 | 0.000 | 0.125 | 0.000 |
| chr6.42511331 | ENSCAFG00000019835 | SORT1 | T/C | *pcadapt* | modifier | 0.250 | 0.350 | 0.300 | 0.167 | 0.167 |
| chr9.25788538 | ENSCAFG00000016947 | FAM117A | T/C | *pcadapt* | modifier | 0.125 | 0.056 | 0.167 | 0.154 | 0.000 |
| chr9.48573827 | ENSCAFG00000019520 | SAPCD2 | G/A | *pcadapt* | modifier | 0.546 | 0.364 | 0.167 | 0.539 | 0.700 |
| chr11.64432892 | ENSCAFG00000002840 | FRRS1L | A/G | *pcadapt* | modifier | 0.167 | 0.556 | 0.333 | 0.269 | 0.100 |
| chr11.64432892 | ENSCAFG00000002847 | EPB41L4B | A/G | *pcadapt* | modifier | 0.167 | 0.556 | 0.333 | 0.269 | 0.100 |
| chr16.5304415 | ENSCAFG00000025529 | TPK1 | A/G | *pcadapt* | modifier | 0.077 | 0.000 | 0.000 | 0.136 | 0.000 |
| chr17.21036425 | ENSCAFG00000004647 | TMEM214 | G/T | *pcadapt* | modifier | 0.115 | 0.364 | 0.250 | 0.417 | 0.100 |
| chr17.21036425 | ENSCAFG00000032246 | *novel gene* | G/T | *pcadapt* | modifier | 0.115 | 0.364 | 0.250 | 0.417 | 0.100 |
| chr17.22859203 | ENSCAFG00000005258 | TOGARAM2 | T/A | *pcadapt* | modifier | 0.042 | 0.091 | 0.000 | 0.231 | 0.250 |
| chr19.38726742 | ENSCAFG00000005161 | DARS | T/C | *pcadapt* | modifier | 0.250 | 0.333 | 0.167 | 0.346 | 0.100 |
| chr24.24026280 | ENSCAFG00000007782 | MYH7B | A/C | *pcadapt* | modifier | 0.083 | 0.045 | 0.100 | 0.250 | 0.000 |
| chr28.40101167 | ENSCAFG00000031153 | *novel gene* | A/G | *pcadapt* | modifier | 0.091 | 0.000 | 0.200 | 0.231 | 0.500 |
| chr28.40101167 | ENSCAFG00000028746 | JAKMIP3 | A/G | *pcadapt* | modifier | 0.091 | 0.000 | 0.200 | 0.231 | 0.500 |
| chr32.17341368 | ENSCAFG00000010071 | PDLIM5 | A/C | *pcadapt* | modifier | 0.450 | 0.042 | 0.000 | 0.208 | 0.083 |
| chr38.21722434 | ENSCAFG00000012569 | CD84 | T/C | *pcadapt* | modifier | 0.208 | 0.091 | 0.100 | 0.083 | 0.083 |
| chr38.22019201 | ENSCAFG00000025205 | ATP1A4 | T/C | *pcadapt* | modifier | 0.154 | 0.050 | 0.250 | 0.308 | 0.000 |
| chr38.22019201 | ENSCAFG00000012557 | NCSTN | T/C | *pcadapt* | modifier | 0.154 | 0.050 | 0.250 | 0.308 | 0.000 |
| chr38.22019201 | ENSCAFG00000012470 | CASQ1 | T/C | *pcadapt* | modifier | 0.154 | 0.050 | 0.250 | 0.308 | 0.000 |
| chr1.55939415 | ENSCAFG00000000868 | SMOC2 | A/G | *GEMMA* | modifier | 0.038 | 0.000 | 0.000 | 0.208 | 0.000 |
| chr2.30074237 | ENSCAFG00000005218 | FBH1 | T/C | *GEMMA* | modifier | 0.000 | 0.182 | 0.167 | 0.000 | 0.000 |
| chr3.55722259 | ENSCAFG00000013671 | EFL1 | C/T | *GEMMA* | modifier | 0.000 | 0.000 | 0.625 | 0.000 | 0.000 |
| chr3.55722259 | ENSCAFG00000013301 | SAXO2 | C/T | *GEMMA* | modifier | 0.000 | 0.000 | 0.625 | 0.000 | 0.000 |
| chr4.75177469 | ENSCAFG00000018909 | MTMR12 | T/C | *GEMMA* | modifier | 0.000 | 0.000 | 0.250 | 0.000 | 0.000 |
| chr5.41739453 | ENSCAFG00000018437 | RAI1 | T/C | *GEMMA* | modifier | 0.250 | 0.111 | 0.083 | 0.577 | 0.000 |
| chr5.6176069 | ENSCAFG00000010304 | ETS1 | G/A | *GEMMA* | modifier | 0.000 | 0.045 | 0.167 | 0.000 | 0.083 |
| chr5.65293686 | ENSCAFG00000019899 | CA5A | G/A | *GEMMA* | modifier | 0.136 | 0.222 | 0.500 | 0.077 | 0.167 |
| chr6.21158415 | ENSCAFG00000017492 | ARHGAP17 | A/G | *GEMMA* | moderate | 0.000 | 0.050 | 0.250 | 0.000 | 0.000 |
| chr7.18162252 | ENSCAFG00000013490 | FAM129A | C/T | *GEMMA* | modifier | 0.045 | 0.042 | 0.000 | 0.500 | 0.000 |
| chr7.62357900 | ENSCAFG00000018132 | TAF4B | C/A | *GEMMA* | modifier | 0.083 | 0.045 | 0.000 | 0.333 | 0.100 |
| chr7.80746043 | ENSCAFG00000013287 | MAPK4 | T/C | *GEMMA* | low | 0.000 | 0.042 | 0.000 | 0.385 | 0.100 |
| chr8.39421602 | ENSCAFG00000016181 | FNTB | A/T | *GEMMA* | modifier | 0.000 | 0.250 | 0.167 | 0.000 | 0.000 |
| chr9.1127695 | ENSCAFG00000005692 | RPTOR | A/T | *GEMMA* | modifier | 0.000 | 0.050 | 0.000 | 0.269 | 0.000 |
| chr9.1635767 | ENSCAFG00000005580 | CCDC40 | G/C | *GEMMA* | modifier | 0.000 | 0.000 | 0.167 | 0.000 | 0.000 |
| chr9.24398370 | ENSCAFG00000016833 | SNX11 | A/G | *GEMMA* | modifier | 0.000 | 0.000 | 0.000 | 0.125 | 0.000 |
| chr9.27812495 | ENSCAFG00000030895 | CA10 | A/G | *GEMMA* | modifier | 0.000 | 0.000 | 0.250 | 0.000 | 0.000 |
| chr9.3889924 | ENSCAFG00000005165 | MGAT5B | A/G | *GEMMA* | modifier | 0.000 | 0.000 | 0.250 | 0.000 | 0.000 |
| chr9.39967470 | ENSCAFG00000018352 | ASIC2 | A/G | *GEMMA* | modifier | 0.000 | 0.000 | 0.200 | 0.000 | 0.000 |
| chr13.34601912 | ENSCAFG00000001181 | TRAPPC9 | T/G | *GEMMA* | modifier | 0.042 | 0.050 | 0.400 | 0.042 | 0.083 |
| chr13.3999486 | ENSCAFG00000000619 | UBR5 | A/G | *GEMMA* | modifier | 0.000 | 0.000 | 0.000 | 0.292 | 0.000 |
| chr15.44910250 | ENSCAFG00000007747 | SLC10A7 | T/A | *GEMMA* | modifier | 0.000 | 0.000 | 0.250 | 0.000 | 0.000 |
| chr17.24572691 | ENSCAFG00000005328 | GALNT14 | G/C | *GEMMA* | modifier | 0.000 | 0.000 | 0.200 | 0.000 | 0.000 |
| chr17.49599329 | ENSCAFG00000008846 | FBXO41 | A/G | *GEMMA* | modifier | 0.000 | 0.000 | 0.000 | 0.208 | 0.000 |
| chr19.43783119 | ENSCAFG00000005403 | LRP1B | T/C | *GEMMA* | modifier | 0.000 | 0.000 | 0.167 | 0.000 | 0.000 |
| chr20.4929652 | ENSCAFG00000004521 | FGD5 | A/G | *GEMMA* | modifier | 0.000 | 0.000 | 0.167 | 0.000 | 0.000 |
| chr21.42167200 | ENSCAFG00000009777 | NAV2 | T/C | *GEMMA* | low | 0.000 | 0.000 | 0.375 | 0.000 | 0.000 |
| chr23.34664941 | ENSCAFG00000007551 | ESYT3 | T/C | *GEMMA* | modifier | 0.000 | 0.091 | 0.583 | 0.192 | 0.250 |
| chr24.26552919 | ENSCAFG00000008832 | RPRD1B | C/A | *GEMMA* | moderate | 0.500 | 0.500 | 0.300 | 0.500 | 0.500 |
| chr24.37322891 | ENSCAFG00000011572 | KCNG1 | A/G | *GEMMA* | modifier | 0.000 | 0.045 | 0.333 | 0.000 | 0.125 |
| chr25.44793783 | ENSCAFG00000011752 | ATG16L1 | C/T | *GEMMA* | modifier | 0.318 | 0.273 | 0.000 | 0.708 | 0.417 |
| chr25.48675988 | ENSCAFG00000012406 | ILKAP | A/G | *GEMMA* | modifier | 0.000 | 0.050 | 0.000 | 0.462 | 0.000 |
| chr28.17335067 | ENSCAFG00000010623 | SORCS3 | A/G | *GEMMA* | moderate | 0.000 | 0.136 | 0.333 | 0.000 | 0.000 |
| chr28.642826 | ENSCAFG00000006576 | WDFY4 | T/A | *GEMMA* | moderate | 0.000 | 0.000 | 0.167 | 0.000 | 0.000 |
| chr31.26259722 | ENSCAFG00000008853 | TIAM1 | A/G | *GEMMA* | modifier | 0.077 | 0.042 | 0.000 | 0.450 | 0.000 |

**Figure S5.** The normalized deviate of homozygosity (F_ND_) calculated for each subpopulation at MHC-linked microsatellite loci. Negative F_ND_ suggests balancing selection, and positive F_ND_ suggests directional selection. Asterisks mark statistically significant values (FDR corrected *p* < 0.011).

**Table S8.** Results from Slatkin’s implementation of the Ewens-Watterson homozygosity test of neutrality performed in *Pypop* for (A) rural and (B) urban subpopulations genotyped at nine MHC-linked microsatellite loci. Significantly negative F_ND_ values (indicated *) suggest balancing selection is acting on that locus.

| **A.** |  | **R_west_ (n=21)** | | | |  | **R_east_ (n=21)** | | | |  | **R_north_ (n=15)** | | | |
| --- | --- | --- | --- | --- | --- | --- | --- | --- | --- | --- | --- | --- | --- | --- | --- |
| **Locus** |  | **F_obs_** | **F_exp_** | **F_nd_** | **p-value** |  | **F_obs_** | **F_exp_** | **F_nd_** | **p-value** |  | **F_obs_** | **F_exp_** | **F_nd_** | **p-value** |
| C2_BF_1 |  | 0.8200 | 0.7716 | 0.3013 | 0.4834 |  | 0.8277 | 0.7740 | 0.3330 | 0.4903 |  | 0.9260 | 0.7487 | 1.1520 | 0.7265 |
| C2_BF_2 |  | 0.8200 | 0.7716 | 0.3013 | 0.4834 |  | 0.8277 | 0.7740 | 0.3330 | 0.4903 |  | 0.9260 | 0.7487 | 1.1520 | 0.7265 |
| CFA12-21 |  | 0.2937 | 0.4258 | -1.0063 | 0.1340 |  | 0.2596 | 0.4258 | -1.2653 | 0.0461 |  | 0.2556 | 0.3965 | -1.2077 | 0.0560 |
| CFA12-19 |  | 0.5918 | 0.7740 | -1.1309 | 0.2010 |  | 0.5000 | 0.7740 | -1.7010 | 0.0099* |  | 0.6089 | 0.7568 | -0.9454 | 0.2766 |
| CFA12-20 |  | 0.5408 | 0.7740 | -1.4476 | 0.1477 |  | 0.5011 | 0.7740 | -1.6940 | 0.0099* |  | 0.5133 | 0.5949 | -0.5234 | 0.3815 |
| DRB1_INTRO2 |  | 0.3980 | 0.4258 | -0.2120 | 0.5053 |  | 0.3197 | 0.4258 | -0.8077 | 0.2281 |  | 0.2444 | 0.3965 | -1.3030 | 0.0319 |
| CFA12-6 |  | 0.3844 | 0.4258 | -0.3156 | 0.4570 |  | 0.5272 | 0.5087 | 0.1237 | 0.6191 |  | 0.6213 | 0.5834 | 0.2507 | 0.5954 |
| GABBR1_DOWN1 |  | 0.2625 | 0.5048 | -1.6369 | 0.0029* |  | 0.3741 | 0.5087 | -0.8983 | 0.2031 |  | 0.2956 | 0.4804 | -1.3511 | 0.0406 |
| CFA12-17 |  | 0.1259 | 0.1892 | -1.1885 | 0.0391 |  | 0.1525 | 0.1859 | -0.6458 | 0.2698 |  | 0.1467 | 0.2440 | -1.4295 | 0.0071* |
| * p-value below the modified FDR corrected threshold 0.011 | | | | |  |  |  |  |  |  |  |  |  |  |  |
|  |  |  |  |  |  |  |  |  |  |  |  |  |  |  |  |
| **B.** |  | **U_east_ (n=32)** | | | |  | **U_west_ (n=11)** | | | |  |  |  |  |  |
| **Locus** |  | **F_obs_** | **F_exp_** | **F_nd_** | **p-value** |  | **F_obs_** | **F_exp_** | **F_nd_** | **p-value** |  |  |  |  |  |
| C2_BF_1 |  | *Monomorphic locus* | | |  |  | *Monomorphic locus* | | |  |  |  |  |  |  |
| C2_BF_2 |  | 0.7997 | 0.7912 | 0.0515 | 0.4531 |  | *Monomorphic locus* | | |  |  |  |  |  |  |
| CFA12-21 |  | 0.2729 | 0.5405 | -1.6437 | 0.0057* |  | 0.3595 | 0.5693 | -1.4496 | 0.0488 |  |  |  |  |  |
| CFA12-19 |  | 0.5488 | 0.7925 | -1.4798 | 0.1427 |  | 0.8347 | 0.7392 | 0.6336 | 0.5594 |  |  |  |  |  |
| CFA12-20 |  | 0.5000 | 0.7912 | -1.7709 | 0.0058* |  | 0.9132 | 0.7392 | 1.1547 | 0.7089 |  |  |  |  |  |
| DRB1_INTRO2 |  | 0.3491 | 0.5405 | -1.1758 | 0.1091 |  | 0.3650 | 0.4427 | -0.6605 | 0.2873 |  |  |  |  |  |
| CFA12-6 |  | 0.5333 | 0.7912 | -1.5684 | 0.1188 |  | 0.5247 | 0.5517 | -0.1979 | 0.4856 |  |  |  |  |  |
| GABBR1_DOWN1 |  | 0.6079 | 0.5405 | 0.4139 | 0.6742 |  | 0.2650 | 0.4427 | -1.5107 | 0.0108* |  |  |  |  |  |
| CFA12-17 |  | 0.2036 | 0.2698 | -0.7493 | 0.2340 |  | 0.2100 | 0.2943 | -1.1172 | 0.0585 |  |  |  |  |  |
| * p-value below the modified FDR corrected threshold 0.011 | | | | |  |  |  |  |  |  |  |  |  |  |  |

**REFERENCES**

Benjamini, Y., and D. Yekutieli. 2001. The control of the false discovery rate in multiple testing under dependency. Annals of Statistics **29**:1165–1188.

Catchen, J., P. A. Hohenlohe, S. Bassham, A. Amores, and W. A. Cresko. 2013. Stacks: An analysis tool set for population genomics. Molecular Ecology **22**:3124–3140.

Luu, K., E. Bazin, and M. G. B. Blum. 2017. pcadapt: An R package to perform genome scans for selection based on principal component analysis. Molecular Ecology Resources **33**:67–77.ÅŒ

Pritchard, J. K., M. Stephens, and P. Donnelly. 2000. Inference of population structure using multilocus genotype data. Genetics **155**:945–959.

Rousset, F. 2008. GENEPOP’007: A complete re-implementation of the GENEPOP software for Windows and Linux. Molecular Ecology Resources **8**:103–106.

Szpiech, Z. A., M. Jakobsson, and N. A. Rosenberg. 2008. ADZE: A rarefaction approach for counting alleles private to combinations of populations. Bioinformatics **24**:2498–2504.

Zhou, X., and M. Stephens. 2012. Genome-wide efficient mixed-model analysis for association studies. Nature genetics **44**:821–4.

Zhou, X., and M. Stephens. 2014. Efficient multivariate linear mixed model algorithms for genome-wide association studies. Nature methods **11**:407–9.
