# Supplementary material for: Urban colonization through multiple genetic lenses: The city‐fox phenomenon revisited
Source: Ecol Evol. 2019 Jan 31;9(4):2046–60. doi: 10.1002/ece3.4898 (PMC6392345; doi:10.1002/ece3.4898)
Supplement: Supplementary file 2 [file ECE3-9-2046-s002.docx]

**Appendix 2. Population Structure Analyses with Intergenic, HWE filtered SNPs**

**Methods.** Please see the methods section “DNA extraction and restriction associated DNA sequencing” for detailed information on laboratory and data processing methods.

Following SNP calling and filtering, we annotated our full SNP data (n=10,149 loci) as intergenic or within an intron, exon, or promoter (within 2 Kb of a transcription start site) as in vonHoldt et al. (2017) using the reference dog CanFam3.1 assembly (Lindblad-Toh et al. 2005). We then created a subsampled dataset of putatively neutral sites to assess whether the full SNP dataset was adequate for analyses of population structure. This dataset consisted of all intergenic SNPs filtered for Hardy-Weinberg equilibrium (HWE) using the --hwe 0.001 flag in *Plink* (Purcell et al. 2007) and contained 5,439 SNPs.

We then ran the analyses described in the “Population structure” section of our study to see whether the full and neutral SNP datasets provided similar results. Please refer to that section for detailed description of analyses run. Briefly, analyses included: (1) genetic assignment tests in *STRUCTURE* (Pritchard et al. 2000)*,* (2) Mantel tests in the *R* package *ecodist* (Goslee & Urban 2007), (3) principal component analysis (PCA) in *flashPCA* (Abraham & Inouye 2014), (4) discriminate analysis of principal components (DAPC) in the *R* package *adegenet* (Jombart et al. 2010), and (5) estimates of pairwise genetic differentiation (F_ST_) and combined private allelic richness.

**Results.** We observed minimal differences between results obtained using our full SNP dataset (n=10,149 loci) and this subdivided dataset of intergenic sites (n=5,439 loci). We therefore retained all SNPs for downstream analysis to maximize the informational content of our data. The figures below contain results using our intergenic and HWE pruned dataset. Please see the complimentary figures included with the main text for direct comparison with results obtained with the full SNP dataset.

**Figure N1.** *STRUCTURE* results for returned by the admixture LOCPRIOR models run with neutral SNPs (n= 5,439 loci). Examination of the mean log probability of *K* supported the presence of one evolutionary cluster. [Complimentary figure with full SNP dataset: Figure S1]

**Figure N2.** The Mantel correlogram showing patterns of spatial genetic structure between pairs of foxes sampled within distance classes of 1000m (n = 50 foxes genotyped at 5,439 SNP loci). Filled circles represent statistically significant (*p* < 0.05) correlations. As observed with the full SNP dataset, the overall Mantel test and Mantel correlogram supported patterns of spatial genetic structure. The highest correlation was observed between pairs of foxes 0-1000m (r=0.205, p=0.001), with the overall Mantel test suggesting moderate correlation across the full range of sampling distances (r=0.245, p=0.001). [Complimentary figure with full SNP dataset: Figure S2]

**Figure N3.** Principal components calculated for 50 foxes across 5,439 SNPs. (A) PC1 plotted against PC2 shows that foxes sampled within the same subpopulation cluster together, with overlap observed between abutting rural and urban subpopulations. (B) When plotted against the Swiss Y-coordinate (northing), PC1 recapitulates the geographic sampling area (inset), thus mirroring the Swiss X-coordinate (easting). As such, PC1 was significantly correlated with the Swiss X-coordinate (easting) in the Spearman’s rank correlation test (*ρ*=0.673, *p*<0.001), with no association detected between PC1 and the Swiss Y-coordinate (northing; *ρ*=0.177, *p=*0.219). [Complimentary figures with full SNP dataset: Figures 2 and S3]

**Figure N4.** Discriminate analysis of principal components (DAPC) revealed (a) overlap between the five sampling locations, with five distinct groups evident and (b) a divide between sampling locations east (R_east_ and U_east_) and west (R_west_ and U_west_) of Lake Zurich and the Limmat River, with R_north_ in the middle. (C) East-west subdivision was somewhat evident in in the major branches of the NJ tree, where each node represents an individual fox colored by sampling locations. Populations east of the barriers (R_east_ and U_east_) clustered closely together, with all other populations more scattered. [Complimentary figure with full SNP dataset: Figure 3]

**Table N1.** As observed with the full dataset, (A) the largest FST value (F_ST_ = 0.0131) occurred between the two urban subpopulations, Ueast+Uwest. The next highest values (F_ST_ = 0.0094, 0.0092) was calculated between urban and rural subpopulations separated by Lake Zurich, the Limmat River, and Zurich’s city center. (B) Private allelic richness for pairwise combinations of subpopulations was highest for adjacent rural-urban subpopulations (Rwest+Uwest=0.0348; Reast+Ueast=0.0326). [Complimentary figures with full SNP dataset: Tables S5 and S6]

| (A) | **F_ST_** | R_east_ | R_north_ | U_east_ | U_west_ |
| --- | --- | --- | --- | --- | --- |
|  | R_west_ | 0.0048 (0.0004) | 0.0053 (0.0005) | 0.0094 (0.0006) | 0.0066 (0.0006) |
|  | R_east_ | . | 0.0049 (0.0005) | 0.0047 (0.0004) | 0.0092 (0.0007) |
|  | R_north_ | . | . | 0.0080 (0.0007) | 0.0069 (0.0008) |
|  | U_east_ | . | . | . | 0.0131 (0.0009) |
|  |  |  |  |  |  |
| (B) | **Private Allele Sharing** | R_east_ | R_north_ | U_east_ | U_west_ |
|  | R_west_ | 0.0232 (0.0012) | 0.0232 (0.0014) | 0.0183 (0.0010) | 0.0348 (0.0018) |
|  | R_east_ | . | 0.0257 (0.0015) | 0.0326 (0.0017) | 0.0227 (0.0014) |
|  | R_north_ | . | . | 0.0205 (0.0012) | 0.0232 (0.0015) |
|  | U_east_ | . | . | . | 0.0197 (0.0012) |

**REFERENCES**

Abraham, G., and M. Inouye. 2014. Fast principal component analysis of large-scale genome-wide data. PLoS ONE **9**:1–5.

Goslee, S. C., and D. L. Urban. 2007. The **ecodist** Package for Dissimilarity-based Analysis of Ecological Data. Journal of Statistical Software **22**. Available from http://www.jstatsoft.org/v22/i07/.

Jombart, T., S. Devillard, and F. Balloux. 2010. Discriminant analysis of principal components: A new method for the analysis of genetically structured populations. BMC Genetics **11**:94.

Lindblad-Toh, K. et al. 2005. Genome sequence, comparative analysis and haplotype structure of the domestic dog. Nature **438**:803–819.

Pritchard, J. K., M. Stephens, and P. Donnelly. 2000. Inference of population structure using multilocus genotype data. Genetics **155**:945–959.

Purcell, S. et al. 2007. PLINK: A tool set for whole-genome association and population-based linkage analyses. American Journal of Human Genetics **81**:559–575.

vonHoldt, B., E. Heppenheimer, V. Petrenko, P. Croonquist, and L. Y. Rutledge. 2017. Ancestry-specific methylation patterns in admixed offspring from an experimental coyote and gray Wolf cross. Journal of Heredity **108**:341–348.
